# 

**Published:** 1880-10

**Authors:** 


**TERMS.—50 Cents a Year. Address The Bistoury, Elmira, N. Y.**

### SUBSCRIPTIONS RECEIVED TO OCTOBER, 1880

NEW YORK—Olean Times, J C Seeley, Dr Geo E Blackman, American Monthly Microscopical Journal, Dr Wm Simpson, Dr J C Dart $2,50, J W Burt $5, J W Henocksburg, J H Barney, Mrs J R Weiss, Dr Johnston, Dr L M Shoemaker, Dr J D Barr, Dr. N D Leisenring, Dr Samuel Flagg, Dr O Henderson, D D Falk, S M Dunn. Dr A Ellwanger, Dr S S Bower, Dr Bliss.

NEW JERSEY—Jas F Norwood, Dr D Curtis, Dr F D Ladd, Dr B B Howes, Mrs A Jones.

PENNSYLVANIA—Jas B Sword, Dr J L Hill $2, Wm Scott, Dr H H Hamilton, D O Ford, Mrs N Tombs, Dr L Keyser, Dr Jacob Luss, Mrs Floyd Hetzel, Dr S D Wynn, Dr N R Liebold, Fletcher Simonds, Dr Hiram Shindel, Dr E Engle, Mrs D R Flurey, Dr. D S Hamilton, Dr F Boyer, Dr Dan Davidson, Dr E F Brown, Dr. A Seicrist, Dr U Hurd.

ALABAMA—Dr D N Moxley $1, Mrs L M Lounsberry.

CONN—Dr C T Stuart, Dr D S Flemming, Dr Geo H Morse, Dr Hiram Ludlow.

KENTUCKY—Dr W S C White, Dr Floyd Ketchum, Dr A Montgomery, Dr D F Banks.

GEORGIA—Dr Frank Spencer, Dr A A Blackburn.

MISSISSIPPI—Dr W C Silliman, Dr H Morensing, Mrs B V English.

WEST VIRGINIA—Dr Geo E Wiley. Dr D Daniels, Mrs Nathan George,

WISCONSIN—Dr Jacob Johnson, Dr Geo Sampson, Henry Daniels.

